# Aberrant stem cell and developmental programs in pediatric leukemia

**DOI:** 10.3389/fcell.2024.1372899

**Published:** 2024-03-27

**Authors:** Rebecca E. Ling, Joe W. Cross, Anindita Roy

**Affiliations:** ^1^ Department of Paediatrics, University of Oxford, Oxford, United Kingdom; ^2^ MRC Molecular Haematology Unit, Weatherall Institute of Molecular Medicine, University of Oxford, Oxford, United Kingdom; ^3^ Department of Haematology, Great Ormond Street Hospital for Children, London, United Kingdom

**Keywords:** leukemia, stem cells, development genes, gene regulation, fetal oncogenes

## Abstract

Hematopoiesis is a finely orchestrated process, whereby hematopoietic stem cells give rise to all mature blood cells. Crucially, they maintain the ability to self-renew and/or differentiate to replenish downstream progeny. This process starts at an embryonic stage and continues throughout the human lifespan. Blood cancers such as leukemia occur when normal hematopoiesis is disrupted, leading to uncontrolled proliferation and a block in differentiation of progenitors of a particular lineage (myeloid or lymphoid). Although normal stem cell programs are crucial for tissue homeostasis, these can be co-opted in many cancers, including leukemia. Myeloid or lymphoid leukemias often display stem cell-like properties that not only allow proliferation and survival of leukemic blasts but also enable them to escape treatments currently employed to treat patients. In addition, some leukemias, especially in children, have a fetal stem cell profile, which may reflect the developmental origins of the disease. Aberrant fetal stem cell programs necessary for leukemia maintenance are particularly attractive therapeutic targets. Understanding how hijacked stem cell programs lead to aberrant gene expression in place and time, and drive the biology of leukemia, will help us develop the best treatment strategies for patients.

## Introduction

Stem cells perform a complex balancing act between self-renewal and differentiation throughout ontogeny. To perform these functions, stem cells proliferate rapidly and repair DNA damage. However, these “stemness” properties present a vulnerability as, if hijacked, they provide cancer cells with the pathways required for growth and survival (Taipale and Beachy, 2001; Hanahan and Weinberg, 2011).

### Hematopoietic stem cells

Human hematopoiesis is a dynamic process beginning at day 18 in the yolk sac (Palis and Yoder, 2001), with definitive hematopoietic stem cells (HSCs) originating in the aorta–gonad–mesonephros from 4 post-conception weeks (pcw) (Tavian and Peault, 2005; Ivanovs et al., 2011). HSCs subsequently migrate to fetal liver (FL), the main site of hematopoiesis until birth (Ivanovs et al., 2017), with contribution from fetal bone marrow (FBM) 10–12 pcw (Charbord et al., 1996; O’Byrne et al., 2019). After birth, BM becomes the sole site of hematopoiesis.

### Fetal HSCs are molecularly and functionally distinct from postnatal HSCs

Fetal HSCs are more proliferative than their postnatal counterparts (Lansdorp et al., 1993; Muench et al., 1994; Bowie et al., 2006; Popescu et al., 2019; Roy et al., 2021) with higher self-renewal capacity (Copley and Eaves, 2013; Copley et al., 2013), better *in vivo* engraftment (Harrison et al., 1997; Holyoake et al., 1999), and a distinct metabolic profile (Manesia et al., 2015). Adult HSCs show a myeloid lineage bias (Benz et al., 2012); additionally, innate lymphoid cells such as B1a B-cells are derived exclusively from embryonic/fetal HSCs (Zhou et al., 2015; Montecino-Rodriguez et al., 2016). These functional differences may be a consequence of distinct developmental gene expression programs.

### Stem cell programs in leukemia

In leukemia, stem cell programs may be inappropriately reactivated or retained and/or co-opted from fetal development. This may be a consequence of some leukemias originating *in utero*, especially in children (Greaves, 2005).

**FIGURE 1:**
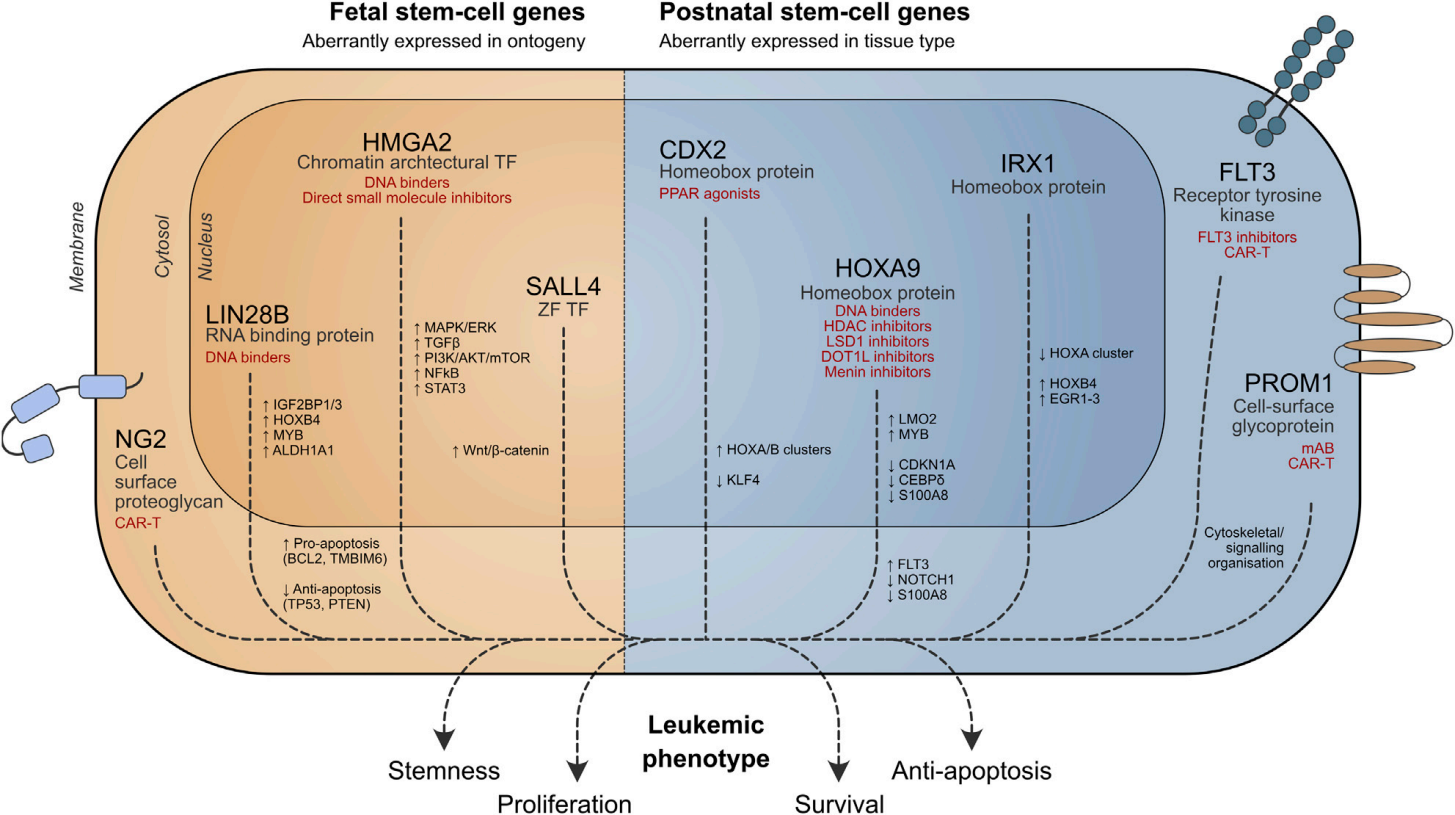
Role of stem cell genes in leukemia cell survival. Potential therapeutic strategies targeting aberrant stem cell-associated pathways are shown in red. TF = transcription factor, ZF = zinc finger.

In this review, we discuss stem cell programs that are aberrantly active in the wrong cellular context (“place”) or stage of ontogeny (“time”) in pediatric leukemia and their potential applications in developing targeted therapies (Figure 1).

## Stem cell programs in the wrong place

### Aberrant stem cell genes in leukemia

Leukemic blasts may exhibit stem cell properties, conferring a more aggressive phenotype. Several stem cell genes are important for leukemia biology (Table 1), and some key examples are discussed below.

**TABLE 1:** Summary of developmental/stem cell genes involved in the pathophysiology of pediatric leukemia.

Stem cell gene	Normal place of and time of expression	Normal function	Aberrant expression and function	Therapeutic potential	Key reference
Transcription factors					
*HOXA9*	Thoraco-caudal neural tube and somites; limb buds	Expression of stemness genes, including *FLT3, MYB, CDK6,* and *RUNX1*	*KMT2A* rearranged ALL and AML (70% of cases) NPM1c-mutated AML	Pre-clinical: co-factor (MEIS1) inhibitors and DNA binders	Vey et al. (2017), Stein et al. (2018), Sonoda et al. (2021), Abedin et al. (2022), Issa et al. (2023), Perner et al. (2023)
	Fetal and adult HSCs		Drives self-renewal and differentiation block	Clinical: HDAC inhibitors (abexinostat and pracinostat); LSD1 inhibitors (bomedemstat); and DOT1L inhibitors (pinometostat); menin inhibitors (revumenib)	
*CDX2*	Embryonic trophectoderm development and gut patterning adult intestinal stem cells	Activation of trophectoderm and intestinal development programs	AML and ALL	Pre-clinical: PPARγ agonists to derepress KLF4	Esmaeili et al. (2021)
			Activates HOX genes and represses KLF4 expression		
*LMO2*	Early yolk sac and fetal definitive hematopoiesis HSCs and angiogenesis	Transcription factor complex with TAL1, GATA1, 2, and 3	Blocks differentiation in T-ALL	Preclinical: macromolecule inhibition of LMO2 is not efficacious as monotherapy	Yamada et al. (1998), Ferrando et al. (2002), Malumbres et al. (2011), Riddell et al. (2014)
			Can induce pluripotency in fibroblasts		
*SALL4*	ESCs, postnatal HSCs	Transcription factor and epigenetic regulator	Pediatric AML	Preclinical	Zhang et al. (2006), Ballerini et al. (2008), Yang et al. (2008), Aguila et al. (2011), Jeong et al. (2011), Ueno et al. (2014), Hodeib et al. (2023)
			Pediatric B-ALL		
			Can induce pluripotency in fibroblasts		
*SOX17*	Fetal HSCs	Transcription factor	Limited evidence in pediatric AML.	Early-phase clinical trials of tazemetostat	Kim et al. (2007), Kormish et al. (2010), Tang et al. (2014)
		Regulates endoderm and hemopoietic differentiation and inhibits Wnt signaling			
*RUNX1 (Hsa21)*	Embryonic/fetal HSCs	Essential for establishment of definitive hematopoiesis	*RUNX1A* isoform important in ML-DS cooperates with GATA1s and miR-125b to upregulate *MYC*	Preclinical MYC inhibitor: MYCi361	North et al. (2002), Gialesaki et al. (2023)
Epigenetic modifiers					
*HMGA 1* and *2*	ESC	Binding to the minor groove of AT-rich DNA sequences alters the chromatin structure to regulate transcription	KMT2A rearranged ALL	Preclinical data: competitive DNA minor groove binders	Roy et al. (2013), Wu et al. (2015), Cinkornpumin et al. (2017), Kumar et al. (2019), Roy et al. (2021)
	Fetal HSC and MPP.		Relapsed B-ALL	Downstream pathway modulation	
				Direct small-molecule inhibition	
*HMGN1 (Hsa21)*	Fetal and adult stem and progenitor cells	De-compacts chromatin and acts in opposition to histone H1	DS-ALL: overexpression promotes PreB-cell expansion and upregulates CRLF2	Pre-clinical: GSK-J4, which targets HMGN1 via inhibition of histone demethylases	Cabal-Hierro et al. (2020), Page et al. (2022)
			AML		
*DNMT3A* and *B*	ESC adult HSCs	DNA methyltransferase to silence HSC regulatory genes	Pediatric AML	Phase 1 trial DNMT inhibitor azacytidine in pediatric r/r AML.	Challen et al. (2011), Liang et al. (2013), Ueno et al. (2014), Liao et al. (2015), Sun et al. (2018)
			DMNT3B included in pLSC6	Ongoing trial in treatment-naive pediatric AML (NCT03164057)	
*EZH2*	ESCs	Component of the PRC2 complex to initiate gene repression	High-risk pediatric AML	EZH2 inhibitor tazemetostat	Ezhkova et al. (2009), Mochizuki-Kashio et al. (2011), D'Angelo et al. (2015), Schäfer et al. (2016), Bond et al. (2018), Italiano et al. (2018)
	Fetal and adult HSCs		B-ALL	Early-phase clinical trial for non-Hodgkin lymphoma and solid malignancies	
			T-ALL		
LSC gene sets					
LSC17	Stem cell gene set	*DNMT3B, GPR56, CD34, SOCS2, FAM30A, ZBTB46, NYNRIN, ARHGAP22, LAPTM4B, MMRN1, DPYSL3, CDK6, CPXM1, SMIM24, EMP1, NGFRAP1,* and *AKR1C3*	AML	Prognostic in adult and pediatric AML cohorts	Ng et al. (2016b)
pLSC6	Stem cell gene set	*DNMT3B, GPR56, CD34, SOCS2, SPINK2,* and *FAM30A*	AML	Prognostic in pediatric AML cohorts	Elsayed et al. (2020)
RNA-binding proteins (RBPs)					
*LIN28B*	ESCs, fetal HSCs, MPP, and LMPP.	Maintains stem cell pluripotency	Pediatric AML	Preclinical data for small-molecule inhibitors including 1,632	Yuan et al. (2012), Copley et al. (2013), Helsmoortel et al. (2016a), Helsmoortel et al. (2016b), Zhang et al. (2016), Wang et al. (2019)
			JMML		
		Acts as negative regulator of *Let-7* micro RNAs (which suppress many oncogenes)	B-ALL		
			Can induce pluripotency		
*IGF2BP1* and *IGF2BP3*	Fetal HSCs, MPP, and LMPP	Posttranscriptional regulation of genes in fetal life	Pediatric AML	Potential for induction of let-7 miRNA to reduce IGF2BP1/3 levels	Alajez et al. (2012), Elagib et al. (2017), Elcheva et al. (2020), Lin et al. (2023), Sharma et al. (2023)
			Pediatric ALL		
miRNA					
*miR-99a/99b*	HSCs	Self-renewal	Pediatric AML and ALL		Moqadam et al. (2013), Zhang et al. (2013), Khalaj et al. (2017)
*miR-125b (Hsa21)*	HSCs	Anti-apoptotic. Confers lymphoid bias to HSCs	Pediatric ALL and Ph + ALL	Prognostic marker in ALL and APML	Klusmann et al. (2010), Ooi et al. (2010), Zhang et al. (2011), Piatopoulou et al. (2017), Alejo-Valle et al. (2022)
			AMKL and APML	Suppresses *ARID3A* and cooperates with GATA1s in ML-DS models	
			ML-DS		
*miR-128a*	HSCs	Maintains stem cell pluripotency	Pediatric AML, ALL, and KMTA2Ar ALL		Georgantas et al. (2007), Mi et al. (2007), De Luca et al. (2017), Malouf et al. (2021)
*miR-155*	HSCs	Bias toward B lymphoid commitment	Pediatric ALL and AML	Synthetic miR-155 phase I T cell lymphoma and ATLL trial (NTC02580552)	Georgantas et al. (2007), Yan et al. (2015), Bayraktar and Van Roosbroeck (2018), Liang et al. (2021)
*miR-181*	HSCs	Regulates HSC differentiation	Pediatric ALL and AML		Zhu et al. (2017), Egyed et al. (2020)
*miR-196b*	HSCs	Increased cell survival and proliferation	Pediatric AML, KMT2Ar ALL, and T-ALL		Popovic et al. (2009), Schotte et al. (2009), Yan et al. (2015)
*24-miRNA*	miRNA signature	mir-20b, mir-223, miR-193, miR-24, miR-128, miR-17, miR-199b, miR-181c, miR-181b, miR-181a, miR-21, miR-222, miR-331, miR-373, miR-708, miR-34b, miR-195, miR-151a, miR-30b, miR-22, let7g, let7i, miR-1290, and miR-9	Pediatric AML	Risk stratification	Esperanza-Cebollada et al. (2023)
Adhesion proteins					
*CD44*	Widespread, including HSCs	Osteopontin/fibronectin/hyaluronan receptor. Adhesion and migration	AML LSC homing to BM	Pre-clinical: CD44 targeting mAb	Jin et al. (2006), Amanzadeh et al. (2018)
			Apoptotic resistance		
Integrins	Widespread, including HSCs	BM microenvironment adhesion and signaling	AML LSC homing to BM	Preclinical	Jäger et al. (2007), Hsieh et al. (2013), Maeda et al. (2015), Darwish et al. (2021), Sudha et al. (2021)
*ITGAM* (CD11b)			Anti-apoptotic signaling	α_4_ targeting mAb	
*ITGAV* (CD51)			Chemoresistance	β2 targeting CAR-T	
*ITGA2/4/6*				Thyrointegrin (α_v_β_3_) targeting drugs	
*ITGB1/2/3*				VLA-4 (α_4_β_1_) targeting peptide	
Selectins	Endothelial cells, leukocytes, and platelets	Permit hematopoietic cell adhesion and rolling	Supports AML LSC survival via PI3K/Akt signaling	Clinical: E-selectin antagonist (uproleselan)	DeAngelo et al. (2022)
*SELE* (CD62E)					
*SELL* (CD62L)		Supports HSC proliferation in the BM niche			
*SELP* (CD62P)					
Other/surface proteins					
*PROM1*	Fetal and adult stem cell populations, including HSCs	Unclear; roles in Wnt signaling, PI3K signaling, and regulating membrane topology	KMT2A-r ALL	Pre-clinical: monoclonal antibody, bispecific CD19/CD133 CAR-T	Wang et al. (2018b), Li et al. (2018), Dai et al. (2020), Vora et al. (2020)
			Cancer stem cells	Clinical: monospecific CD133 CAR-T	
*CRLF2 (Hsa21)*	Hematopoiesis	Dimerizes with IL7RA to form the receptor for thymic stromal lymphopoietin	Overexpressed and rearranged in	Tyrosine kinase inhibitors	Bagashev et al. (2022), Tian et al. (2023)
	Fetal lymphopoiesis		DS-ALL BCR-ABL like ALL	Preclinical: TSLPR CAR-T; IB7/CD3 bispecific antibody	
*DYRK1A (Hsa21)*	Neural tissues	Serine/threonine kinase. Required for normal lymphoid maturation	ML-DS	Preclinical: small-molecule DYRK1A inhibitor, EHT1610	Malinge et al. (2012), Thompson et al. (2015), Bhansali et al. (2021), Carey-Smith et al. (2024)

Italicsed words are gene names.

### Homeobox genes

Homeobox genes form a group of transcription factors (TFs) with 235 functional genes in humans (Holland et al., 2007).

#### HOXA9

The largest class of homeobox genes, the *HOX* genes (Holland, 2013), play important roles in hematopoiesis, such as development and maintenance of HSCs (Magnusson et al., 2007; Ramos-Mejía et al., 2014). HOXA9 can reprogram tissues to a hematopoietic fate (Ng E. S. et al., 2016; Sugimura et al., 2017).

Over 50% of AML cases overexpress *HOXA9*, which correlates with poor survival (Drabkin et al., 2002; Andreeff et al., 2008; Tholouli et al., 2012). HOXA9 directly binds target genes along with PBX3/MEIS1 (Wong et al., 2007; Li et al., 2016), upregulating oncogenes such as *FLT3, LMO2*, and *MYB*(Huang et al., 2012; Collins et al., 2014). HOXA9 also suppresses apoptotic/differentiation factors, promoting leukemia cell survival and maintaining a more stem-like state (Agrawal-Singh et al., 2023).

*HOXA9* plays a key role in *KMT2A*-rearranged (*KMT2A-r*) leukemia (Faber et al., 2009; Orlovsky et al., 2011), mediated by binding of the KMT2A fusion protein to *HOX* gene promoters (Milne et al., 2002; Milne et al., 2005) and recruitment of DOT1L H3K79 methyltransferases (Bernt et al., 2011; Kerry et al., 2017). Dysregulated *HOXA9* expression is also seen in *NPM1c*-mutated AML (Brunetti et al., 2018; Uckelmann et al., 2023).

#### CDX2

*CDX2* of the ParaHox family of homeobox genes (Brooke et al., 1998) is not normally expressed in hematopoietic cells and inhibits the hematopoietic potential of murine embryonic stem cells (ESCs) (McKinney-Freeman et al., 2008).

Over 90% of AML cases overexpress *CDX2* (Chase et al., 1999; Scholl et al., 2007; Rawat et al., 2008), and ectopic expression of *Cdx2* confers oncogenic properties to murine HSCs (Rawat et al., 2004; Scholl et al., 2007). *CDX2* overexpression studies suggest upregulation of *HOX* genes as the oncogenic mechanism (Rawat et al., 2008), although at least one alternative pathway is through direct suppression of KLF4 (Faber et al., 2013).

Aberrant *CDX2* expression is also frequently seen in ALL (Riedt et al., 2009; Thoene et al., 2009) and confers a poor prognosis (Yasuda et al., 2022).

#### *IRX* genes

The *Iroquois* genes (*IRX1-6*) belong to the TALE group of homeobox genes. *IRX1* is required for normal development of kidney and neural tissues (Alarcón et al., 2008; Freese et al., 2014) but not expressed in most hematopoietic precursors (Nagel et al., 2022). *IRX3* and *IRX5* are not expressed in hematopoiesis.

In *KMT2A-r* infant ALL, *IRX1* or *HOXA9* expression defines two distinct subgroups (Stam et al., 2010; Symeonidou and Ottersbach, 2021). *IRX1* prevents KMT2A::AFF1 from activating *HOXA9* expression; instead, expression of *HOXB4* causes persistence of HSC factors (Kuhn et al., 2016). *IRX1*+(*HOXA*-) *KMT2A-r* ALL has a poorer prognosis (Agraz-Doblas et al., 2019; Isobe et al., 2022).

In AML, *IRX1/3/5* is aberrantly expressed (Nagel and Meyer, 2022). In AML cell lines, expressions of *IRX1* and *IRX3/5* are mutually exclusive with opposing effects on *GATA1/2* activity (Somerville et al., 2018).

### PROM1

Prominin-1 (PROM1/CD133) is a membrane pentaspan glycoprotein, identified in the mouse neuroepithelium (Weigmann et al., 1997) and human HSCs (Miraglia et al., 1997; Yin et al., 1997). Its function remains enigmatic; and its role in cytoskeletal organization is postulated. In solid organ malignancies, PROM1 regulates extracellular vesicle formation and release (Rappa et al., 2013; Kang et al., 2019; Zhao et al., 2020).

*PROM1* is expressed in many cancers (Reya et al., 2001; Singh et al., 2004; Bao et al., 2006; Ferrandina et al., 2009), including leukemia, where the expression is associated with poor prognosis (Tolba et al., 2013). It is a common feature of *KMT2A-r* leukemias and essential for survival of KMT2A::AFF1 cell lines (Godfrey et al., 2021; Wang L.-l. et al., 2022).

### Leukemic stem cells

In addition to inappropriate expression of stem cell genes, there may be very rare leukemic stem cells (LSCs) within a leukemia that can propagate disease in serial transplantation (Lapidot et al., 1994; Hope et al., 2004; Hong et al., 2008). This concept is well-established in AML (Dick, 2008), but less so in ALL (Cox et al., 2009). AML LSCs share the key feature of self-renewal with normal HSCs alongside similarities in gene expression (Sachs et al., 2020), immunophenotype (e.g., CD133+(Tolba et al., 2013), CD123+(Testa et al., 2002)), and metabolism (Manesia et al., 2015).

A stem cell signature (LSC17) has been used to risk stratify adult (Ng S. W. K. et al., 2016) and some pediatric AMLs (Duployez et al., 2019), where it confers a worse prognosis. A six-gene pediatric AML signature (pLSC6) (Elsayed et al., 2020) was derived from a cohort of 163 pediatric AMLs (Table 1). The adult and pediatric signatures share five genes (*DNMT3B, GPR56, CD34, FAM30A/KIAA012,* and *SOCS2*), although with different gene weightings, and pLSC6 includes a further unique gene, *SPINK2*.

Single-cell RNA-seq identified LSC clusters in pediatric AML; these LSC markers require functional validation (Zhang Y. et al., 2023). Fetal-specific genes (*HMGA2)* have been identified in a rare HSC-like fraction of *KMT2A-r* infant leukemia (Chen et al., 2022), and *IGF2BP1* maintains LSC by regulating *HOXB4, MYB*, and *ALDH1A1* in pediatric leukemia cell lines (Elcheva et al., 2020).

## Aberrant stem cell gene expression in ontogeny

### Fetal stem cell programs in pediatric leukemia

Pediatric leukemias [and solid malignancies such as neuroblastoma (Molenaar et al., 2012)] may exhibit properties of fetal HSCs. This may represent a fetal cell of origin or indicate reactivation of fetal programs (Monk and Holding, 2001; Sharma et al., 2022; Solé et al., 2022). Either way, the inappropriate expression of fetal genes is important for cancer biology. Leukemic blasts, especially LSCs (Somervaille et al., 2009), show fetal-specific gene expression profiles (Wu et al., 2015; Helsmoortel et al., 2016b; Elcheva et al., 2020; Bai et al., 2021; Tran et al., 2021). Down syndrome (DS)-associated leukemias and juvenile myelomonocytic leukemia (JMML) are two examples resulting from perturbation of fetal HSPCs.

Trisomy 21 causes global perturbation of fetal hematopoiesis, with increased phenotypic HSCs and megakaryocyte–erythroid progenitors (MEPs), as well as a marked skew to erythropoiesis with a concomitant decrease in B-lymphopoiesis (Chou et al., 2008; Roy et al., 2012; Jardine et al., 2021). DS fetal HSPCs and stromal cells also display increased inflammatory signatures (Jardine et al., 2021). Chromosome 21 (Hsa21) stem cell genes dysregulated/overexpressed in DS include transcription factor GABPA, which affects HSC maintenance/differentiation (Yu et al., 2011), and chromatin modifier *HMGN1*.

Children with DS have an increased risk of AML and ALL (Hasle et al., 2000). Mutations in the megakaryocyte–erythroid transcription factor GATA1 in fetal life lead to transient abnormal myelopoiesis (TAM) in the fetal/neonatal period (Roberts et al., 2013; Wagenblast et al., 2021). Additional mutations, most commonly in the cohesin complex genes (Labuhn et al., 2019), are required for myeloid leukemia of DS (Roberts and Izraeli, 2014). DS-ALL also likely stems from perturbed lymphopoiesis, which begins *in utero*, and is characterized by *CRLF2/*TSLPR overexpression in 50% and *JAK2* mutations in 20% (Li et al., 2023). Key Hsa21 genes important for leukemogenesis are listed in Table 1.

JMML, an HSC-derived leukemia (Cooper et al., 2000; Louka et al., 2021), has a fetal molecular profile (Roy et al., 2021; Hartmann et al., 2023). As the oncogenic hit probably occurs in fetal HSCs, developmental stem cell programs are hijacked for leukemogenesis.

Fetal oncogenes relevant to stem cell activity and implicated in pediatric leukemia act via a diverse range of mechanisms and are discussed below (Figure 1).

### Fetal genes with transcription factor activity implicated in pediatric leukemia

*SCL/TAL1* was originally identified as overexpressed in T-ALL (Ferrando et al., 2002). Ablation of the gene causes embryonic death (Porcher et al., 2017), but it is dispensable for adult HSCs (Mikkola et al., 2003).

*LMO2* is essential for fetal hematopoiesis (Yamada et al., 1998). *LMO2* expression is seen in a subset of pediatric T-ALL (Ferrando et al., 2002) and B-ALL (Malumbres et al., 2011). In gene therapy trials for SCID, two patients developed T-ALL through off-target activation of *LMO2* (Hacein-Bey-Abina et al., 2003).

*SALL4* is expressed in ESCs (Zhang et al., 2006), but downregulated postnatally with only low-level expression in HSCs (Gao et al., 2013). It is aberrantly expressed in pediatric AML (Ballerini et al., 2008) and ALL (Den Boer et al., 2009; Ueno et al., 2014), with overexpression conferring poor prognosis (Harvey et al., 2010; Jeong et al., 2011).

### Fetal genes important for post-transcriptional regulation

The bulk of post-transcriptional control is exerted by RNA-binding proteins (RBPs). Some RBPs such as LIN28B and insulin-like growth factor 2 mRNA-binding proteins (IGF2BP1/IGF2BP3) have a fetal expression pattern, a role in stem cell biology (Copley and Eaves, 2013; Degrauwe et al., 2016; Zhang et al., 2016) and pediatric leukemia.

### LIN28B

LIN28B has wide-ranging physiological roles in fetal tissues; however, the expression after birth is limited to the placenta and testis (Uhlén et al., 2015). In hematopoiesis, FL HSCs have the highest expression of *LIN28B* (Roy et al., 2021), and expression in postnatal cells can reactivate fetal-like erythropoiesis (Lee et al., 2013; Basak et al., 2020) and B-lymphopoiesis (Yuan et al., 2012). *LIN28B* can reprogram somatic cells to induce pluripotency (Zhang et al., 2016); however, this can be oncogenic in other contexts (Wuputra et al., 2020).

The main action of LIN28B is to prevent maturation of let-7 miRNAs (Rybak et al., 2008; Viswanathan and Daley, 2010). LIN28B also directly stabilizes many mRNAs in conjunction with IGF2BP1/IGF2BP3. In murine B progenitors, this includes *Pax5* and *Arid3a*, thereby driving fetal B-lymphopoiesis (Wang et al., 2019).

*LIN28B* is frequently aberrantly expressed in cancers, including leukemia (Viswanathan et al., 2009; Balzeau et al., 2017). Suppression of *let-7* miRNAs by LIN28B leads to de-repression of oncogenes (*MYC, RAS, MYB,* and *ARID3A*) and epigenetic regulators, *HMGA2* and *CBX2* (Wang D. et al., 2022).

A meta-analysis showed that 7.5% of pediatric leukemias express *LIN28B* (Helsmoortel et al., 2016b). Aberrant *LIN28B* expression defines a poor prognosis subgroup in JMML (Helsmoortel et al., 2016a), where *H19*, a fetal oncogene (Matouk et al., 2014), is stabilized in the presence of LIN28B (Helsmoortel et al., 2016b). AML in children <3 years has higher levels of *LIN28B* (and *IGF2BP1/3*) expression than in children >3 years (Bolouri et al., 2021). Although *LIN28B* has predominantly been reported to have a pro-leukemic role in AML (Zhou et al., 2017), one study on a murine KMT2A::MLLT3 AML model suggests that *LIN28B* abrogates perinatal leukemia development (Eldeeb et al., 2023). Given >50% of human neonatal leukemias are of myeloid lineage, these findings seem counterintuitive, although it is possible that neonatal AML arises from LIN28B negative progenitors. Given the role of LIN28B in fetal B-lymphopoiesis, it may also be important for ALL initiation or maintenance.

### *IGF2BP1* and *IGF2BP3*

IGF2BP1 and IGF2BP3 are important for fetal organogenesis and are expressed in FL HSCs, but not in adult HSCs (Wang D. et al., 2022). Induction of *IGF2BP3* in adult HSCs induces a fetal-type output (Palanichamy et al., 2016; Wang et al., 2019).

IGF2BP1 and IGF2BP3 have been linked to leukemia, as well as solid malignancies, and are often co-expressed with LIN28B (Elcheva et al., 2020; Tran et al., 2021). The mechanism of action for IGF2BP3 in oncogenesis is segregation of mRNA transcripts from the cytoplasmic RNA-induced silencing complex, including the let-7 miRNA family.

IGF2BP1 is linked to pediatric AML and *ETV6/RUNX1* B-ALL, while IGF2BP3 is linked to AML, KMT2A::AFF1 ALL, and BCR::ABL1 ALL (Stoskus et al., 2011; Palanichamy et al., 2016; Elcheva et al., 2020; Zhang et al., 2022). IGF2BP1 supports an LSC phenotype in AML (Elcheva et al., 2020). In AML cell lines, knockdown of IGF2BP3 leads to reduced cell proliferation in an N6-methyladenosine (m6A)-dependent fashion (Zhang et al., 2022). Depletion of the murine paralog IGF2BP3 increases the latency of leukemia in murine models of KMT2A::AFF1 AML (Tran et al., 2021).

### Fetal genes important for epigenetic regulation

#### *HMGA1* and 2

*HMGA1* and *HMGA2* are fetal oncogenes affecting epigenetic regulation. The HMGA family encodes proteins with AT hooks which interact with DNA to alter the chromatin architecture. These genes have much lower expression in adult tissues than in the fetal counterparts (Kumar et al., 2019; Roy et al., 2021), and *HMGA1* can promote a pluripotent state (Shah et al., 2012). *HMGA2* is expressed mainly in fetal HSC/MPP and influences both differentiation and proliferation of stem cells (Battista et al., 2003; Li et al., 2007; Copley et al., 2013), as well as promoting long-term *in vivo* reconstitution by cord blood CD34^+^ cells (Kumar et al., 2019).

Reactivation of *HMGA1* and *HMGA2* has been demonstrated in a wide range of malignancies (Huso and Resar, 2014; Mansoori et al., 2021) including leukemia (Efanov et al., 2014). *HMGA1* expression has been linked to risk of relapse in pediatric B-ALL (Roy et al., 2013). In pediatric and adult AML, high expression of *HMGA2* is linked to poor prognosis, and knockdown of the gene has induced differentiation in primary blasts (Marquis et al., 2018; Tan et al., 2018). *HMGA2* induces T-ALL in a Eμ-HMGA2 transgenic mouse (Efanov et al., 2014).

### microRNAs in leukemia

Aberrant expression of microRNAs (miRNAs) specific to fetal life and stem cell compartment (O'Connell et al., 2010) is implicated in pediatric leukemia (Grobbelaar and Ford, 2019; Gaur et al., 2020) (Table1). Pediatric AML can be risk-stratified by a 24-miRNA signature (Esperanza-Cebollada et al., 2023). Eight of these have target genes within the pLSC6 signature and includes *let-7* miRNAs (known repressors of oncogenes), with lower *let-7g/let-7i* expression in high-risk AML. One of the pLSC6 genes (*FAM30A)* is an lncRNA. Signatures based on lncRNA differentiate pediatric leukemia subtypes, but do not inform prognosis (Buono et al., 2022).

## Targeting aberrant stem cell programs in leukemia for therapy

The inappropriate expression of stem cell genes, while conferring survival advantage to leukemic cells, can also render them dependent on specific proteins or pathways, and thus vulnerable to targeted disruption. Fetal stem cell genes are the most attractive targets as they are not expressed in healthy postnatal tissues, ameliorating concerns about off-target effects. Genes expressed in leukemic and healthy postnatal stem cells present more of a challenge. However, excessive leukemic reliance on the aberrant pathway, the so-called “oncogenic addiction,” can generate a therapeutic window, whereby leukemic cells can be killed while sparing normal stem cells. Potential targeting strategies are summarized in Table 1. Specific approaches relating to stem cell genes discussed in this review are explored below.

### Small-molecule inhibitors

Many stem cell genes code for TFs or other DNA-binding proteins, considered “undruggable,” owing to their intrinsically disordered nature. Recent improvements in screening methods have identified HMGA2-binding compounds, including the antimicrobials sumarin and ciclopirox (Huang et al., 2019; Su et al., 2020) and MEIS1/2 inhibitors (Turan et al., 2020).

An alternative approach employs small molecules that bind the minor groove of the TF cognate sequence. DNA binders of this type can inhibit HOXA9 (Depauw et al., 2019), with *in vitro* activity against *HOXA9*-dependent cells lines (Sonoda et al., 2021). Similarly, netropsin and trabectedin demonstrate antitumor activity in *HMGA2+* neoplasia. Treatment with both drugs shows a synergistic anti-proliferative effect in infant ALL cell lines (Wu et al., 2015). Other approaches to target *HMGA2* include targeting downstream pathways such as G2M transition (Moison et al., 2022) and PI3K/Akt/mTOR (Tan et al., 2016).

TF function can also be impaired by preventing their expression. *HOXA9* transcription is dependent on DOT1L-mediated H3K79 methylation in *KMT2A-r* leukemia. DOT1L has been successfully targeted with small molecules, pinometostat/EPZ5676 (Basavapathruni et al., 2014; Waters et al., 2016). Early-phase clinical trials suggest modest activity against *KMT2A-r* leukemia (Stein et al., 2018). Newer DOT1L inhibitors with oral availability and improved pharmacokinetics have been developed (Stauffer et al., 2019; Perner et al., 2020).

HOXA9 is also dependent on the scaffold protein menin for expression in KMT2A-r leukemias (Yokoyama et al., 2005) and *NPM1c*-mutated AML (Kühn et al., 2016). The small-molecule revumenib prevents the interaction of menin with its target proteins. Revumenib induces remission in 30% of relapsed/refractory leukemia patients (Issa et al., 2023), although mutations in *MEN1* can lead to drug resistance (Perner et al., 2023).

Direct inhibition of CDX2 has not yet been possible; however, the observation that PPARγ signaling restores KLF4 expression offers a potential therapeutic route to partially opposing CDX2 activity. PPARγ agonists are toxic to *CDX2* overexpressing leukemia cell lines *in vitro* (Faber et al., 2013; Esmaeili et al., 2021).

### Targeting RNA-binding proteins

Like DNA-binding proteins, small-molecule inhibition of RBPs is difficult, although recent high-throughput approaches have generated candidates (Wu, 2020). The most promising LIN28(A/B) inhibitor is C1632, which targets *LIN28B +* cell lines both by disruption of LIN28B–let-7 interaction (Franses et al., 2020; Zhang Q. et al., 2023; Shahab et al., 2023) and in Ewing sarcoma by disruption of the interaction between *EWS-FLI1* mRNA and LIN28B (Keskin et al., 2020). Other molecules such as LI71 bind the cold shock domain and have efficacy against *LIN28B +* cancer cell lines (Wang L. et al., 2018).

Small-molecule inhibitors of IGF2BP1 and IGF2BP3 are at the preclinical stage. BTYNB destabilizes oncogenic transcripts by disrupting the IGF2BP1–mRNA association (Müller et al., 2020; Jamal et al., 2023; Sharma et al., 2023). Combining menin inhibitors with depletion of IGF2BP3 impairs cell growth and increases differentiation of KMT2A::AFF1 leukemia (Lin et al., 2023).

Another potential strategy to boost let-7 miRNA expression, thus inhibiting several oncogenes (Cinkornpumin et al., 2017), has yet to be applied in leukemia.

### Immune effector cell therapy

Stem cell markers as targets for immunotherapy: The cell surface marker PROM1 is a highly attractive target for immunotherapy. A CD19/CD133 tandem CAR-T (Li et al., 2018) and CD19/133 bispecific CAR-iNKT (Ren et al., 2023) show efficacy *in vivo* against *KMT2A-r* cell lines. However, valid concerns about stem cell toxicity when targeting CD133 in patients have been raised (Bueno et al., 2019). Preclinical testing and early-phase trials using CD133-CAR-T in solid malignancies (Wang et al., 2018b; Dai et al., 2020; Vora et al., 2020) revealed no BM aplasia and only transient, reversible hematological toxicities. Longer-term follow-up and assessment will be required to confirm the safety of CD133 targeting. CAR-T directed against NG2 has shown promise in mobilizing leukemic blasts and rendering them more sensitive to chemotherapy in mouse models (Lopez-Millan et al., 2019). Anti-CD117 CAR-T therapy shows preclinical efficacy (Myburgh et al., 2020) but also eliminates healthy HSCs, necessitating novel approaches such as terminating “safety switches” (Magnani et al., 2023). Both CAR-T (Wang et al., 2018c) and CAR-NK (Mansour et al., 2023) cells have been used to target FLT3. Anti-CD123 CAR-T therapy has been used in early-stage clinical trials, appearing safe and potentially effective (Yao et al., 2019; Wermke et al., 2021).

Immunotherapy is particularly attractive in DS-associated leukemias where conventional treatments cause significant toxicities. In DS-ALL patients, CD19-, CD22-, and TSLPR-directed immunotherapies could yield promising results (Bagashev et al., 2022; Laetsch et al., 2023).

## Discussion

Stem cells possess unique properties allowing the expansion, self-renewal, and differentiation required for tissue homeostasis. These programs are frequently co-opted by leukemias, where they provide growth and survival advantages. Although there is renewed interest in reprogramming adult HSCs to become more “fetal-like”, the potential of fetal stem cell genes to also promote oncogenesis must be considered.

Understanding stem cell programs in leukemia, including oncofetal genes, is vital to disentangling the biology of leukemias, including treatment resistance/relapse, and identifying mechanisms vulnerable to novel targeted therapies.
